# Classification and evaluation of the environment of the professional nursing practice in a teaching hospital*

**DOI:** 10.1590/1518-8345.4339.3376

**Published:** 2020-10-19

**Authors:** Dilzabeth Margot Imata Yanarico, Alexandre Pazetto Balsanelli, Renata Cristina Gasparino, Elena Bohomol

**Affiliations:** 1 Universidade Federal de São Paulo, Escola Paulista de Enfermagem, São Paulo, SP, Brazil.; 2 Scholarship holder at the Programa de Alianças para a Educação e a Capacitação (PAEC OEA-GCUB)/UNIFESP, Brazil.; 3 Universidade Estadual de Campinas, Faculdade de Enfermagem, Campinas, SP, Brazil.

**Keywords:** Working Environment, Health Facility Environment, Nursing Care, Professional Practice, Working Conditions, Hospitals, Teaching, Ambiente de Trabalho, Ambiente de Instituições de Saúde, Cuidados de Enfermagem, Prática Profissional, Condições de Trabalho, Hospital de Ensino, Ambiente de Trabajo, Ambiente de Instituciones de Salud, Atención de Enfermería, Práctica Profesional, Condiciones de Trabajo, Hospitales de Enseñanza

## Abstract

**Objective:**

to classify and evaluate the environment of the professional nursing practice in a teaching hospital.

**Method:**

a cross-sectional study conducted with 188 nurses from a teaching hospital in the state of São Paulo, SP, Brazil. A questionnaire with sociodemographic and professional data and the Brazilian version of the Practice Environment Scale were used to classify and evaluate the environment of the professional nursing practice. Data was analyzed using Student’s t-test, analysis of variance, Mann-Whitney, and Kruskal-Wallis tests with a significance level of 5% (p<0.05). The internal consistency of the instrument was evaluated using Cronbach’s alpha.

**Results:**

the score’s mean for the Brazilian version of the Practice Environment Scale was 2.54, and the participants considered two of the five subscales as unfavorable for the practice, namely: subscale 1, “Nurse Participation in Hospital Affairs” (2.37), and subscale 4, “Staffing and Resource Adequacy” (2.23).

**Conclusion:**

the environment of the professional nursing practice has been classified as mixed, being evaluated with favorable conditions for the nursing practice, but the participation and involvement of nurses in hospital matters and the adequacy of resources to provide quality care need improvements.

## Abstract (pt)

**Objetivo:**

classificar e avaliar o ambiente da prática profissional de enfermeiros em hospital de ensino.

**Método:**

estudo transversal com 188 enfermeiros de um hospital de ensino do estado de São Paulo, SP, Brasil. Utilizou-se um questionário com dados sociodemográficos e profissionais e a *Practice Environment Scale* - versão brasileira para classificar e avaliar o ambiente de prática profissional dos enfermeiros. Os dados foram analisados com os testes t de Student, análise de variância, Mann-Whitney e Kruskal-Wallis com nível de significância de 5% (p<0,05). A consistência interna do instrumento foi avaliada pelo alfa de Cronbach.

**Resultados:**

a média do escore para a *Practice Environment Scale* - versão brasileira foi de 2,54 e os participantes consideraram duas das cinco subescalas desfavoráveis para a prática: subescala 1 “Participação dos enfermeiros na discussão dos assuntos hospitalares” (2,37) e a subescala 4 “Adequação da equipe e de recursos” (2,23).

**Conclusão:**

o ambiente de prática profissional dos enfermeiros foi classificado como misto avaliando-o com condições favoráveis ao exercício da enfermagem, porém a participação e envolvimento dos enfermeiros nos assuntos hospitalares e a adequação de recursos para prestar assistência com qualidade necessitam de melhorias.

## Abstract (es)

**Objetivo:**

clasificar y evaluar el entorno de la práctica profesional de los enfermeros en un hospital universitario.

**Método:**

estudio transversal con 188 enfermeros de un hospital universitario en el estado de São Paulo, SP, Brasil. Se utilizó un cuestionario con datos sociodemográficos y profesionales y la versión brasileña de la *Practice Environment Scale* para clasificar y evaluar el entorno de práctica profesional de los enfermeros. Los datos se analizaron mediante pruebas t de Suden, análisis de varianza, Mann-White y Kruskal-Wallis con un nivel de significación del 5% (p <0,05). La consistencia interna del instrumento fue evaluada por el alfa de Cronbach.

**Resultados:**

el puntaje promedio para la - versión brasileña fue 2,54 y los participantes consideraron que dos de las cinco subescalas eran desfavorables para la práctica: subescala 1 “Participación de enfermeros en la discusión de los asuntos del hospital” (2,37) y subescala 4 “Dotación de personal y los recursos” (2,23).

**Conclusión:**

el entorno de la práctica profesional de los enfermeros se clasificó como mixto, habiéndose considerado favorables las condiciones para el ejercicio de la enfermería, sin embargo, la participación y el involucramiento de los enfermeros en los asuntos hospitalarios y la dotación de recursos para brindar atención de calidad requieren mejoras.

## Introduction

The work done by the health professionals is continuous and permanent to ensure the provision of care to the patients. It is composed of a multi-professional team and in it we find a nursing team, whose main focus is direct care for the patients^(^ 1 ^)^.

The work developed by the nursing team in providing care in the health services is considered important due to its big proportion regarding other health professionals; however, it is fundamental that they are satisfied with the work they do and that they have an adequate work environment^(^ 2 ^)^.

The evaluation of the environment quality is an important indicator to sustain the work of the nurse who, as the team’s leader, needs to be aware of the two pillars that organize their practice, in order to ensure the quality of the care provided^(^ 3 ^)^.

For nursing, the work environment is known as the organizational characteristics that facilitate or limit the professional practice and, when they are facilitating, they can benefit the individuals and the quality of care^(^ 4 ^-^ 5 ^)^.

A widely known instrument to measure these characteristics is the Nursing Work Index (NWI), developed in the 1980s to evaluate the satisfaction at work and the quality of care between different hospitals^(^ 5 ^)^. In 2000, the NWI was enhanced, resulting in the Nursing Work Index Revised (NWI-R), with the objective of synthesizing the presence of certain characteristics at the work environment^(^ 3 ^,^ 6 ^)^. Thus, the instrument was validated and adapted to several cultures in the world, including Brazil, aiming to evaluate the characteristics of the work environment in the hospitals^(^ 7 ^)^. The instrument’s latest review culminated in the development of the Practice Environment Scale-Nursing Work Index (PES-NWI), whose objective is to verify the presence of characteristics that favor the professional nursing practice^(^ 5 ^)^.

The PES-NWI was considered a useful tool to measure the nurses’ work environment; therefore, it is recommended by organizations in the United States of America. For instance, the National Quality Forum (NQF) has endorsed it as a preferential measure in the nursing practice, and the Joint Commission (JC) has included the PES-NWI as a nursing care effectiveness indicator^(^ 4 ^,^ 8 ^-^ 9 ^)^. So, the PES-NWI was widely adapted and validated in several international contexts^(^ 10 ^)^. By virtue of being a briefer instrument, having methodological rigor, and leading to appropriate measures, the scale was validated in 2017 for the Brazilian culture^(^ 11 ^)^.

Thus, evaluating the environment of the professional nursing practice is important not only to favor quality care to the patient but also to promote a favorable atmosphere to the health team. Then, it gets clear that few Brazilian studies used the PES to evaluate the environment of the professional nursing practice. In addition, they only investigated specific hospital settings^(^ 8 ^)^.

Moreover, it should also be noted that the teaching hospitals must pay attention to this issue, as it is in this environment that all the care actions will be offered to the patient, besides the training of new professionals^(^ 12 ^)^. Hence, it is justified to evaluate the environment of the professional nursing practice in a teaching hospital, considering all its units to diagnose what needs to be enhanced, aiming to provide nurses with better work conditions and thus raise the quality of care provided to the patients. And, in addition, to contribute with this investigative topic considering the Brazilian scene^(^ 8 ^)^.

In view of this, this research sought to answer the following question: What are the characteristics of the environment of the professional nursing practice who work in a teaching hospital? The objective of the study was to classify and evaluate the environment of the professional nursing practice working in a teaching hospital.

## Method

A cross-sectional and descriptive study of a quantitative approach, conducted in a teaching hospital in the city of São Paulo, SP, Brazil. This service is a very important place as it is a university hospital. Currently, it has 800 beds and serves the most diverse medical specialties. It hosts medical internship, an uni-, and multi-professional programs. Data collection was performed between September and October 2018. As the inclusion criterion, a working time of six months or more as a nurse was considered; and, as the exclusion criterion, nurses that were on vacation or leave in the data collection period. A pilot test was carried out for sample calculation with 17 participants that were selected out of a 479-nurse population who answered the research questionnaire. We used the *Dimensionamento Amostral* - DIMAM^®^ program with a confidence level of 95% and a sample error of 0.10, resulting in a minimum sample of 178 participants. 225 questionnaires were distributed randomly but, to comply with the inclusion and exclusion criteria, 188 questionnaires answered by the nurses were used. The participants from the pilot test were excluded. These nurses that composed the sample worked in the following hospital units: inpatient, emergency, intensive therapy, surgical center, specialized outpatients, and other settings such as hospital infection control service, and diagnostic Medicine.

For the data collection, the nurses received a two-section questionnaire, the first one with questions on sociodemographic and professional information, and the second one with the Brazilian version of the PES^(^ 11 ^)^.

The sociodemographic and professional variables were the following: age, gender, number of children, marital status, time since graduation, experience time as a nurse in the institution, unit of work, time of experience at the unit, current position as a nurse, work shift, working hours, and type of work contract.

The second section, the Brazilian version of the PES, was composed of 24 items and 5 subscales. Subscale 1, Nurse Participation in Hospital Affairs (five items), demonstrates the role and value of the nurse in the wide hospital context; Subscale 2, Nursing Foundations for Quality Care (seven items), stresses a nursing philosophy aimed at high care quality standards; and subscale 3, Leadership and Support of Nurses (five items), focuses on the nursing manager role in the institution, encompassing key skills that a nurse in this position needs to have; subscale 4, Staffing and Resource Adequacy (four items), describes the need for an adequate team and the support from resources in order to promote quality care; and subscale 5, Collegial Nurse-Physician Relations (three items), characterizes the positive work relations between nurses and physicians^(^ 5 ^,^ 11 ^)^.

The answer options range from one to four points, using a Likert type scale: Totally disagree (one point); Disagree (two points); Agree (three points), and Totally agree (four points)^(^ 11 ^)^, that is, the higher the score, the higher the presence of favorable characteristics to the development of the nursing activities.

For the evaluation of the subscales, the mean of the sum of the participants’ answers is calculated. With values above 2.5, the environment is considered favorable for the professional practice^(^ 11 ^)^. Thus, with scores above 2.5 in a subscale, the environment is considered unfavorable; scores above 2.5 in two or three subscales represent a mixed environment for the practice; and, with scores above 2.5 in four or five subscales, the environment is considered to be favorable for the professional nursing practice^(^ 5 ^,^ 10 ^)^.

The questionnaires were distributed in the different work shifts at all the hospital units. So, the collected data were analyzed by the R software, version 3.6.0.

Descriptive statistics were used to obtain the arithmetic mean and standard deviation of the continuous variables and absolute and relative frequencies of the categorical variables. The Shapiro-Wilk test was used to test the normality of the variables and the parametric and non-parametric tests were used to verify the existence of relations between the study variables. The Student’ test was used for normal distribution of two groups; Variance Analysis (ANOVA), for data with a normal distribution of more than two groups; the Man-Whitney test, for abnormal data of two groups, and Kruskal-Wallis was used for data with abnormal distribution with more than two groups, followed by Dunn’s post-test, when differences were found. A 5% statistical significance level was considered (*p-value*<0.05) between the different subscales and study variables.

The internal consistency of the instrument was evaluated using Cronbach’s alpha. The interpretation of the instrument’s reliability ranges from 0 to 1, in which values above 0.75 are considered reliable^(^ 13 ^)^.

The research project was approved by the participating health institution and had the authorization of the Research Ethics Committee, considering the ethical standards according to Resolution 466/2012 (opinion No. 2,877,378). After approval, the participants received and signed the Free and Informed Consent Form (FICF).

## Results

The mean age of the participants was 40.1 years old, (SD=9.3); 171 (91%) were female; 78 (41.5%) had no children, and 90 (47.9%) were married. Regarding the professional variables, the mean time of experience in the profession was 12.8 years (SD=9.4); experience in the institution: 13.8 years (SD=8.6); 82 (43.6%) worked at inpatient units with a mean time of experience 6.5 years (SD=6.3); 180 (95.7%) worked in the assistance area; 72 (38.3%) worked in the morning shift; 75 (39.9%) worked 36 hours per week, and 136 (72.3%) were hired under employment laws regime.

Regarding the reliability of the instrument, the participants’ responses in relation to the Brazilian version of the PES and the subscales are presented in Table 1.

**Table 1:** Reliability, arithmetic mean, median, and standard deviation of the Brazilian version of the PES*: general and subscales. São Paulo, SP, Brazil, 2018

PES*	Cronbach's Alpha	Mean	Median	SD^†^
General	0.90	2.54	2.48	0.50
1. Nurse Participation Hospital Affairs	0.80	2.37	2.40	0.70
2. Nursing Foundations for Quality Care	0.76	2.55	2.57	0.58
3. Leadership and Support of Nurses	0.83	2.66	2.80	0.72
4. Staffing and Resource Adequacy	0.67	2.23	2.25	0.66
5. Collegial Nurse-Physician Relations	0.73	3.00	3.00	0.62

* PES = Practice Environment Scale - Brazilian version;

† SD = Standard deviation

Thus, the statistically significant differences found when relating the professional variables to the overall score and to the subscales of the Brazilian version of the PES were presented in Table 2.

**Table 2:** Relationship between the professional variables and the scores of the PES* subscales - Brazilian version, of the nurses working in a teaching hospital. São Paulo, SP, Brazil, 2018

Variables	General		Subscales									
			1		2		3		4		5	
	AM^†^	p^‡^	AM^†^	p^‡^	AM^†^	p^‡^	AM^†^	p^‡^	AM^†^	p^‡‡^	AM^†^	p^‡^
Experience as a nurse												
< 5 years	2.6	0.42^§^	2.5	0.26^§^	2.6	0.64^||^	2.8	0.11^§^	2.2	0.30^§^	2.9	0.61^§^
5 to 10 years	2.4		2.2		2.5		2.5		2.1		3.0	
11 to 15 years	2.6		2.5		2.6		2.7		2.4		3.1	
> 15 years	2.6		2.4		2.6		2.7		2.3		3.0	
Experience in the institution												
< 5 years	2.6	0.15^§^	2.4	0.23^§^	2.5	0.27^||^	2.7	0.06^§^	2.2	**0.04** ^§^	3.0	0.69^§^
5 to 10 years	2.4		2.2		2.4		2.4		2.1		2.9	
11 to 15 years	2.7		2.5		2.6		2.8		2.5		3.1	
> 15 years	2.6		2.4		2.6		2.7		2.2		3.0	
Work unit												
Hospitalization unit	2.6	0.18^§^	2.4	0.66^§^	2.6	0.17^||^	2.7	0.13^||^	2.3	**0.02** ^§^	3.0	0.24^§^
Emergency Room	2.4		2.3		2.3		2.9		1.8		2.8	
Intensive Care Unit	2.4		2.2		2.4		2.4		2.2		3.0	
Surgical Center	2.4		2.3		2.5		2.7		1.9		2.4	
Ambulatory	2.7		2.5		2.7		2.8		2.4		3.1	
Others	2.5		2.3		2.6		2.6		2.1		3.2	
Experience at the unit												
< 5 years	2.6	**0.03** ^§^	2.4	0.11^§^	2.6	0.19^||^	2.7	**0.03** ^§^	2.2	0.11^§^	2.9	0.08^§^
5 to 10 years	2.4		2.2		2.4		2.4		2.1		3.0	
11 to 15 years	2.8		2.6		2.7		2.9		2.6		3.4	
> 15 years	2.5		2.3		2.6		2.6		2.1		3.0	
Current position as a nurse												
Assistance	2.5	0.75^§^	2.4	0.38^§^	2.5	0.81^||^	2.6	0.24^§^	2.2	0.46^§^	3.0	0.13^§^
Coordinator	2.5		2.4		2.5		2.9		2.1		2.7	
Manager	2.7		3.0		2.8		3.1		1.9		2.5	
Work shift												
Morning	2.5	0.31^§^	2.3	0.14^§^	2.5	0.36^||^	2.7	0.18^||^	2.2	0.37^§^	3.0	0.68^§^
Afternoon	2.6		2.5		2.6		2.7		2.4		3.0	
Evening	2.5		2.3		2.6		2.5		2.2		3.0	
Morning/Afternoon	2.7		2.7		2.7		2.8		2.2		3.1	
Weekly working hours												
30 hours	2.6	0.42^§^	2.4	0.83^§^	2.6	0.59^||^	2.7	0.30^||^	2.3	**0.004** ^§^	3.0	**0.03** ^§^
36 hours	2.6		2.4		2.5		2.7		2.3		3.0	
40 hours	2.4		2.3		2.5		2.6		2.0		3.0	
Others	2.6		2.4		2.5		2.3		2.5		3.4	
Employment contract												
Single Legal Regime	2.6	0.42^¶^	2.5	0.25^¶^	2.6	0.14^||^	2.6	0.73^¶^	2.3	0.23^¶^	3.0	0.70^¶^
Consolidation of Labor Laws	2.5		2.4		2.5		2.7		2.2		3.0	

* PES = Practice Environment Scale - Brazilian version;

† AM = Arithmetic mean;

‡ p = Significance level;

§ Kruskal-Wallis;

|| ANOVA;

¶ Mann-Whitney

Thus, Dunn’s post-test found differences in the scores for subscale 3 between the “time of experience in the unit” variable from 5 to 10 years with <5 years and 11 to 15 years categories. Of subscale 4 with the “time of experience as a nurse in the institution” variable from 11 to 15 years with groups of <5 years and from 5 to 10 years. There were also differences from the same subscale with the emergency and outpatient work units, and with the weekly working hours, with different scores for all the groups. By the same post-test differences were found between the scores of subscale 5 and the “weekly working hours” variable, with the “others” category having higher mean scores than the other groups.

## Discussion 

The instrument’s mean score showed to be lower than that of studies that evaluated the practice in countries like the United States, China, and Turkey^(^ 14 ^-^ 16 ^)^. Considering that some components favoring the development of nursing activities need financial investment to get enhancements, the economic inequality between the countries may be a possible explanation to this finding^(^ 17 ^)^, besides, in Brazil, public hospitals suffer from the underfunding by the system^(^ 18 ^)^.

Still about the United States, we can often observe favorable environments for the nursing practice and this happens due to the fact that many American hospitals already have the Magnet^®^ Recognition Program, a certificate that was initiated in the 1980s by the American Academy of Nursing (AAN) and which recognizes institutions that perform an excellent nursing practice by means of a transforming leadership, empowerment of the nursing structure and picture, performing of a model practice, implementation of improvements, and outcome enhancements^(^ 19 ^)^.

On the other hand, a national research study carried out in hospitals similar to the one in this study, that is, a public and teaching one, has revealed similar results to the ones presented here^(^ 20 ^)^. We highlight with regret that, in the Brazilian scenario, although we have only one research study published until now considering the whole hospital, the environment’s characteristics of the hospitals funded by the Unified Health System (*Sistema* Único *de Saúde*, SUS) are more precarious when compared to those of the private hospitals^(^ 20 ^)^ and, also, to the international literature^(^ 14 ^-^ 15 ^,^ 21 ^)^.

The underfunding of the public hospitals^(^ 18 ^)^ can contribute to features such as support services, continuing education programs, staff sizing, and development in the career not being adequately present in institutions that are fully funded by the SUS.

It is worth noting that, even before the realities experienced by different health institutions in Brazil and in the world, it is important that the managers commit themselves to keep a work environment that is favorable for the nursing practice, since improvements in the environment collaborate for positive results both in the patients (safety improves), and in the professionals (reduced burnout and intention to leave the job)^(^ 14 ^-^ 16 ^,^ 22 ^)^.

In the classification of the environment, the studied institution presented a mixed work environment, as subscale 1, “Nurse Participation in Hospital Affairs”, and subscale 4, “Staffing and Resource Adequacy”, presented unfavorable scores for the professional nursing practice. Other hospitals in the national^(^ 20 ^)^ and international^(^ 23 ^)^ contexts also had their environments classified as mixed and it is interesting to highlight that, even in favorable environments, the “Staffing and Resource Adequacy” subscale always received the lowest score^(^ 14 ^-^ 15 ^,^ 21 ^)^.

This inadequacy of support services and of the number of nursing professionals for the provision of excellent care may be related to financial difficulties that strike the institutions, but also to a devaluation of the work of these professionals. Internationals bodies point out to a need for appreciation of the nursing role, for these professionals, who stay 24 hours beside the patients, are underpaid and victims of gender and work condition inequalities^(^ 24 ^)^.

Table 2 showed the statistically significant differences between “the time of experience as a nurse in the institution”, “work unit”, and “working hours” variables with subscale 4, “Staffing and Resource Adequacy”. Thus, the younger nurses, who worked at the emergency room and had a weekly workload of 40 hours, had more unfavorable perceptions in this subscale which mainly addresses the staff sizing issue. The burden related to the overcrowded services in the emergency rooms and extensive working hours might have contributed to this perception^(^ 25 ^)^.

A statistical difference was also found between time of experience at the unit and subscale 3, “Leadership and Support of Nurses”; considering that the nurses who worked there for between five and ten years present an unfavorable opinion. Thus, a number of studies reveal an association between longer working time in the institution and less labor involvement^(^ 1 ^)^.

Finally, there was a statistical difference between the weekly working hours and subscale 5, “Collegial Nurse-Physician Relations”. Although the category “others” in the working hours variable reached a higher mean than the others, all showed favorable results in this subscale. International^(^ 14 ^-^ 15 ^,^ 21 ^,^ 23 ^)^ and national^(^ 20 ^)^ studies also evidenced that the relationships between these professionals are one of the best-evaluated dimensions when the characteristics of the environment are evaluated and this result is indeed satisfactory, to the extent that the conflicting relationships favor flaws in communication, which contributes to events^(^ 26 ^)^.

This way, considering the scarcity in the Brazilian literature on the evaluation of features that favor the professional nursing practice, this study has contributed to the mapping of environments where nursing develops its activities so that it can be continuously broadened. Besides, the managers in the studied institution will be able to implement improvements in the subscales that received unfavorable scores, especially those that do not require a financial investment.

As a limitation of this study, we can highlight the fact that it was conducted only with nurses. Considering that the great majority of the nursing team is composed of technicians and nursing auxiliaries, new research studies involving this population and in different scenarios must be developed in order to have a picture of the reality of Brazilian nursing practice environments.

## Conclusion

The environment of the professional nursing practice was classified as mixed and was evaluated with favorable conditions to the nursing practice, but the participation and involvement of the nurses in the hospital’s matters and the resources’ adequacy to provide a quality care need improvements.

The results of the research suggest attention from the management to the nursing team since a mixed work environment may lead to a better or worse work condition. Therefore, there is a need for a look towards actions for improvement of the working conditions regarding the time and opportunity that the managers can give to the nurses to participate in the discussion of hospital matters and decisions. It is necessary to empower nurses by believing in their potential to transform health practices. In addition, resources’ adequacy improvements are recommended in order to provide quality care assistance.
